# Analysis of payments to GI physicians in the United States: Open payments data study

**DOI:** 10.1002/jgh3.12401

**Published:** 2020-08-21

**Authors:** Venu Gopala Reddy Gangireddy, Rajan Amin, Kevin Yu, Praveen Kanneganti, Swathi Talla, Amarnath Annapureddy

**Affiliations:** ^1^ United Hospital Center West Virginia University Bridgeport West Virginia USA; ^2^ Department of Internal Medicine University of Texas Health Science Center at Houston Houston Texas USA; ^3^ Baptist Memorial Hospital Memphis Tennessee USA; ^4^ Section of Cardiovascular Medicine, Department of Internal Medicine Yale School of Medicine New Haven Connecticut USA

**Keywords:** affordable care act, compensation, gastroenterology, payment

## Abstract

**Background and Aim:**

The purpose of this study was to review and analyze the nature of industry payments to gastroenterology and hepatology (GI) physicians.

**Methods:**

We conducted a retrospective study of open payments (OP) data for the year 2017. Payments to individual physicians were aggregated using a unique physician profile identification number. General payments to Centers for Medicare and Medicaid Services regions were also analyzed. The nature of financial transactions in general payments was reported overall and per physician payment. Research, ownership, and general payments were aggregated and analyzed by drug/device companies.

**Results:**

During the study period, more GI physicians received contributions in the form of general payments compared to ownership or research payments. A small percentage of physicians received contributions greater than $100 000. The most frequent contributions were for food and beverages. Only 10 manufacturers made about 71% ($43 271 938) of general payments.

**Conclusions:**

We found that only a small number of GI physicians received a significant portion of industry payments. A large portion of those payments came from drug or device companies. The impact of these payments on gastroenterologists needs to be examined further.

## Graphical abstract

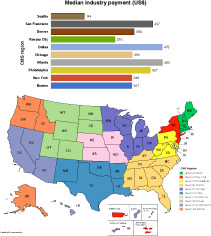

## Introduction

Medical professionalism has been the cornerstone of medical practice, dating back many decades, at least to the inception of the Hippocratic oath.^1^ The modern health‐care system includes various components beyond just the physician and the patient. Other components include the government, pharmaceutical companies, marketing agencies, insurance companies, and other stakeholders.

The financial aspect of health care is currently the driving factor in terms of research, access to care, and prescription patterns. Federal agencies, such as the National Institutes of Health and Centers for Disease Control and Prevention, have been the cornerstone of funding biomedical research for years, but industry‐sponsored research is now outpacing federal funding.^2^ Pharmaceutical and medical device companies are both, directly and indirectly, investing in various forms of funding to reach physicians.^3^ As a result, many ethical questions are now arising from physician ties to the pharmaceutical industry. Initially, voluntary disclosure of financial conflict of interest was thought to be enough, but research studies have shown inadequate compliance with such disclosures.^4^, ^5^, ^6^

Recognizing the importance of transparency in health care, the U.S. government passed “The PPSA (Physician Payments Sunshine Act)” as part of the Affordable Care Act.^7^ The PPSA requires manufacturers of medical products to disclose to Centers for Medicare and Medicaid Services (CMS) any payments of value that were made to teaching hospitals or individual physicians. The data collection was started in 2013 and first reported in 2014. Recent studies on Open Payments have shown the significant financial interaction between the medical industry and physicians in various specialties.^8^, ^9^, ^10^, ^11^, ^12^, ^13^, ^14^, ^15^, ^16^ This also brought to light the inaccuracies of financial disclosure statements and conflicts of interests, thus advocating for more stringent enforcement of disclosure policies.^6^, ^17^, ^18^ Few studies briefly reviewed payments to gastroenterology and hepatology (GI) physicians, but a detailed analysis has not yet been conducted.^9^, ^11^, ^19^ The rationale of our study is to address this gap and to bring into focus the nature of industry payments to gastroenterologists and hepatologists.

## Objective

The main objective of our study is to review the industry payments to GI physicians. The secondary objective is to analyze the nature of payments and key industry sponsors to GI physicians.

## Methods

We conducted a retrospective study of open payments (OP) data for the year 2017^20^ OP is a federally run program that collects and publicly reports information about financial relationships between the health‐care industry, individual physicians, and academic hospitals. Further details on OP data can be found in detail online through the government website.^7^

Payments made to the individual physician and teaching hospitals were broadly categorized into research payments, ownership interests, and general payments. Research payments include payments made with regard to research protocols or research agreements. Stocks, bonds, and partnership shares in the related company or group‐purchasing organizations are included in ownership interest. General payments include all other payments.^7^

We collected data only for allopathic/osteopathic GI physicians. Payments to individual physicians were aggregated using a unique physician profile identification number. General payments to CMS regions were also analyzed.^7^ The nature of financial transactions in general payments was reported overall and per physician payment. Definitions and additional details for the nature of each type of financial transactions were reported as a Table S1, Supporting information. Research, ownership, and general payments were aggregated and analyzed by drug/device companies. All statistical analyses were performed using SPSS software version 23 (IBM Corp, Armonk, NY).

## Results

### *General characteristics*

**Graph 1:**
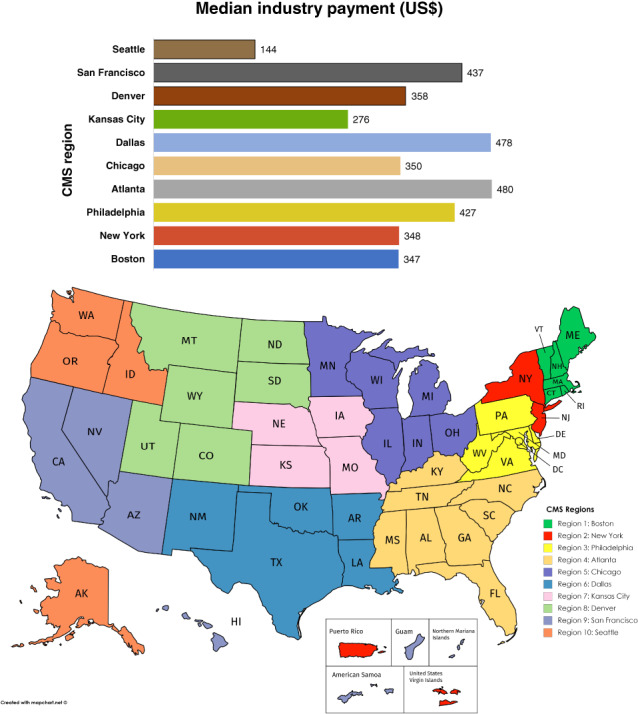
Open payments by Centers for Medicare and Medicaid Services region. (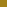), Seattle; (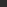), San Francisco; (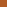), Denver; (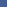), Kansas City; (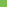), Dallas; (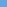), Chicago; (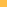), Atlanta; (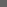), Philadelphia; (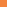), New York; (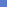), Boston.

The General Payments group had the highest number of recipients and total aggregated payments compared to the Research or Ownership groups (12 743/$61 169 576 *vs* 185/$3 442 931 *vs* 16/$3 442 931, respectively). The median payments were higher for the Ownership group compared to the General group or Research group ($25 000 *vs* $398 *vs* $2905, respectively) (Table 1). Only a small number of GI physicians received contributions of more than $100 000. Most of the physicians received less than $1000 (median per physician payment $202) in the General group, between $1000 and $9999 (median per physician payment $4502) in the Research group, and $10 000–99 999 in the Ownership group (median per physician payment $30 653) (Table S1). The Atlanta area CMS region received the highest median general payment of $480, while the Seattle region received the lowest median payment of $144 (Graph 1).

**Table 1:** General characteristics

	Characteristics		Total
General payments	No. of recipients		12 743
	No. of payments		388 164
	Value of payments, US $		61 169 576
	Median annual per physician (IQR)	No. of payments	14 (3‐44)
		Payments, US $	398 (115‐1127)
Research payments	No. of recipients		185
	No. of payments		438
	Value of payments, US $		1 607 286
	Median annual per physician (IQR)	No. of payments	2.37 (1–2)
		Payments, US $	2905 (435–6636)
Ownership	No. of recipients		16
	No. of payments		19
	Value of interest US $		3 442 931
	Total amount invested US $		3 341 663
	Median annual per physician (IQR)	No. of payments	1
		Value of interest	25 000 (5579–50 000)
		Total amount invested, US $	25 000 (5579–50 000)

IQR, interquartile range.

### *Method of compensation*

Most of the payments were made for compensation for services other than consulting ($24 495 320) (Table 2). The most frequent contributions were made for food and beverages (12 411). Median per physician payment was the highest for royalty or license ($48 565).

**Table 2:** Nature of identified general payment to physicians

Payment type	Total ($)	Median ($)	No. of physicians	Per physician ($)	
				Median ($)	Median no. of payments (IQR)
Charitable contribution	750	750	1	750	1
Entertainment	14 432	43	96	92	1 (1–2)
Gift	47 396	15	166	87	1
Education	667 585	12.51	1366	34	1 (1–2)
Grant	754 921	1160	57	1250	1
Current or prospective ownership or investment interest	1 133 398	5020	6	5020	1
Honoraria	1 325 606	1800	236	3251	2 (1–3)
Compensation for serving as faculty or as a speaker	3 374 625	3000	278	6600	2 (1–4)
Royalty or license	5 407 668	34 212	27	48 565	4 (1–4)
Travel and lodging	6 237 434	179	1946	1024	4 (2–10)
Food and beverage	7 479 193	14.66	12 411	335	13 (3‐43)
Consulting Fee	10 231 246	2500	972	4425	2 (1–4)
Compensation for service other than consulting	24 495 320	2250	1296	8700	5 (2–10)

### *Payments by industry*

A total payment of $61 169 576 was made by 393 drug/device companies to GI physicians in the General Payments group, $1 607 286 by 32 companies in the Research group, and $33 416 63 (invested amount) by 12 companies in the Ownership group for the year 2017 (Tables 3, 4, and 5 and Table S2). Only 10 manufacturers made about 71% ($43 271 938) of the general payments. GI Supply had the highest aggregated payments ($2 772 000) in the Ownership group, Eli Lilly in the Research group ($298 751), and AbbVie in the General Payments group ($9 560 796). While the payments in each category varied widely by drug/device company, compensation for service, consulting fee, education, food, and travel are the most frequently compensated categories. Linzess by Allergan was the highest‐paid sponsored medication ($5 769 700) (Graph S1, Supporting information).

**Table 3:** Top 10 industry payments in general payments section

Name of the manufacturer	Aggregate amount of payments	No. of physicians	Compensation for service	Consulting fees	Education	Food	Travel	Others
AbbVie	9 560 796	84 238	6 068 523	998 782	3865	1 581 180	899 030	9416 (grant)
Gilead sciences	8 086 431	4025	4 611 713	1 782 684	2269	599 700	975 985	114 081 (grant)
Allergan	6 339 060	5886	4 798 113	15 896	1291	806 360	717 380	20 (gift)
Valeant pharmaceuticals North America	4 263 539	5827	55 200	176 667	1602	767 910	408 989	2 732 650 (compensation for serving) 7130 (gift) 113 392 (royalty)
Takeda*	3 151 475	5424	1 754 707	663 621	43 633	417 663	271 851	
Merck Sharp & Dohme Corporation	3 102 648	4127	1 770 328	626 770	1405	274 970	429 175	
Cook*	2 443 557	595	185 650	51 100	42 400	66 388	41 795	10 605 (compensation for serving) 261 101 (grant) 1 784 517 (royalty)
Johnson & Johnson*	2 366 415	5558	1 137 900	365 048	30 731	518 745	313 991	
Braintree Laboratories	2 012 637	1962	90 002	42 533		56 130	2562	2900 (grant) 1 818 511 (royalty)
Boston Scientific Corporation	1 945 380	1969	726 008	97 272	1638	221 232	232 474	388 552 (current or prospective) 278 204 (grant)

* Takeda (Takeda Development Center America; Takeda Pharmaceutical Company; Takeda Pharmaceuticals America; Takeda Pharmaceuticals Puerto Rico; Takeda Pharmaceuticals U.S.A.); Cook (Cook Incorporated; Cook Medical LLC; Wilson Cook Medical Incorporated); Johnson and Johnson (Janssen Biotech, Inc.; Janssen Global Services, LLC; Janssen Pharmaceuticals, Inc; Janssen Products, LP; Janssen Research & Development; Janssen Scientific Affairs, LLC; Johnson & Johnson Health Care; Johnson & Johnson Surgical Vision.

**Table 4:** Industry payments in ownership section

	Frequency	Aggregated payments
GI Supply, Inc.	1	2 772 000
Saphena Medical, Inc.	2	215 658
Endogastric Solutions, Inc	2	124 998
Atlas Spine, Inc.	2	75 000
Cardiosolutions, Inc.	3	50 015
The North Carolina Mutual Wholesale D	1	36 306
Bio2 Medical, Inc.	1	25 000
Medimetriks Pharmaceuticals, Inc.	1	25 000
Romark Laboratories, LC	1	11 600
Vertebral Technologies, Inc.	1	3572

**Table 5:** Industry payments in research section

	Aggregate	Number
Eli Lilly and Company	298 751	78
Gilead Sciences Inc	217 735	46
ChiRhoClin, Inc.	188 475	11
AbbVie, Inc.	168 610	168
Valeant Pharmaceuticals North America	143 184	4
UCB Biosciences Inc.	127 415	15
Olympus Corporation	114 469	5
SANOFI US SERVICES INC.	76 515	1
Vanda Pharmaceuticals Inc.	75 000	7
EndoStim, Inc.	70 677	12

## Discussion

The advent of the PPSA has shed light on the role of industry contributions to physicians of various subspecialties. This study characterizes the nature of industry payments made to gastroenterologists using the Open Payments Database.

We found that general payments made up a more substantial proportion of industry contributions to physicians compared to research and ownership payments. It is similar to other studies that examined industry payments to various subspecialties.^10^, ^11^, ^15^ In a study by Pathak *et al*. looking into payments to pediatric orthopedic surgeons, 0.07% of payments were for research, and 0.2% were for ownership, with the remaining total going toward general payments.^15^, ^29^ These findings may be due to specific preferences in spending patterns by different companies. It could also be a result of the wide variety of payments that are included within the general payments category.

Our study shows that a small subset of GI physicians received the most significant industry contributions, greater than $100 000. This is similar to several other studies.^21^, ^22^, ^23^ Some of the earlier studies have inferenced this disproportionality to targeting influential leaders in the industry, who are more likely to have an impact within their respective fields.^24^, ^25^, ^26^ In a study by Brauer *et al*., the investigators examined the nature of payments to otolaryngologists. They noted that there was a disparity in total payments to the top 40 *versus* the remaining 417 otolaryngologists.^27^ The investigators further evaluated the data and discovered that the top 40 received payments from more companies and for more studies compared to those not in the top 40. Although our study examines the general nature of industry payments to GI physicians, this does not specifically differentiate the payments made between top and bottom earners. This could be pursued in future studies.

Regarding regional differences in compensation, we found that the Atlanta area received the highest payments, whereas the Seattle region received the lowest. These differences may reflect regional variations in physician compensation. They may also reflect differences in physician attitudes toward accepting industry payment. Further studies would be required to better understand the geographic distribution of payments.

The findings of this study reveal from whom GI physicians receive compensations and the types of compensation by using the data that are made available through the Open Payments Database. However, this study's findings are primarily descriptive and do not analyze the potential influence of payments on GI physician clinical practice patterns and delivery of care. Several prior studies found that physician behavior can be influenced by compensation from industries.^28^ Drug/Device companies made the largest contribution of payments by industry. Previously Tringale *et al*. noted that interventionalists (cardiologist, gastroenterologists, and anesthesiologists) recieved highest median payment compared to others (surgeons, primary care physicians and non‐interventional specialists).^11^ Additional studies are warranted to understand whether and how compensation from industries directly affects GI physician practice patterns.

The main limitation of this study derives from the data that were available through the Open Payments Database. Parts of the data lacked the granularity to allow for precise analysis. For example, while the data were able to categorize the many different types of compensation in the general payment's category, the most significant number of payments was labeled compensation for services other than consulting. It is unclear what these compensations are based on the data available through the Open Payments Database. Because it has been shown that behavior can be influenced by compensation from industries, it may be valuable to characterize what types of compensations are utilized in this broad group of payments to allow for more transparency of the interactions between industry and GI physicians.

Moreover, GI physicians are a broad group that encompasses general GI physicians, hepatologists, advanced endoscopists, etc. Our study and others like it have shown that industries tend to target a select group of GI physicians, and one can only speculate who these physicians are. Stratification of the different subspecialties may allow for follow‐up analyses on the effect of compensation by industries and physician practice patterns.

## Conclusion

Our study took the data from the Open Payments Database from 2017 and teased out the different methods of compensation that were similar to other medical specialties, such as cardiology, orthopedic surgery, and otolaryngology. A significant portion of the payments was made to a small number of GI physicians, and a large part of the payments came from drug or device companies. Additional studies on the influence of industry payments and changes in practice patterns of GI physicians will help achieve the goal of the PPSA for transparency in the relationship between industry and physicians.

## Supporting information


**Graph S1** Top 10 highest paying sponsored medications.

**Table S1** Characteristics per physician.
**Table S2** General payments: Top 10 aggregated payments per physician by drug or device manufacturers.
