# Supplementary material for: Analysis of payments to GI physicians in the United States: Open payments data study
Source: JGH Open. 2020 Aug 21;4(6):1031–6. doi: 10.1002/jgh3.12401 (PMC7731803; doi:10.1002/jgh3.12401)
Supplement: Supplementary file 1 — Graph S1 Top 10 highest paying sponsored medications. [file JGH3-4-1031-s001.docx]

**Supplemental Graph 1. Top 10 Highest Paying Sponsored Medications**
