# Supplementary material for: Analysis of payments to GI physicians in the United States: Open payments data study
Source: JGH Open. 2020 Aug 21;4(6):1031–6. doi: 10.1002/jgh3.12401 (PMC7731803; doi:10.1002/jgh3.12401)
Supplement: Supplementary file 2 — Table S1 Characteristics per physician. Table S2 General payments: Top 10 aggregated payments per physician by drug or device manufacturers. [file JGH3-4-1031-s002.docx]

**Supplemental Table 1: Characteristics per physician**

|  | **Per Physician payments (US $)** | **No.of Recipients (%)** | **No.of Payments** | **Total Value of payments (%)** | **Median annual per physician (IQR)** | |
| --- | --- | --- | --- | --- | --- | --- |
|  |  |  |  |  | **No.of payments** | **Payments, US $** |
| **General Payments** | <1000 | 9173 (72) | 136070 | 2 752 017 (4.5) | 6 (2-23) | 202 (80 – 475) |
|  | 1000-9999 | 2583 (20.3) | 167364 | 6 668 886 (10.9) | 66 (29-91) | 1623 (1236 – 2948) |
|  | 10000-99999 | 873 (6.9) | 62725 | 27 231 089 (44.5) | 57 (30-103) | 22 680 (14 526 - 40773) |
|  | ≥ 100000 | 114 (0.9) | 22005 | 24 517 585 (40.1) | 183 (117 – 248) | 163 595 (130 336 – 219 400) |
| **Research Payments** | <1000 | 63 (34.6) | 63 | 17996 (1.2) | 1 (1-2) | 115 (20—500) |
|  | 1000-9999 | 93 (49.7) | 232 | 414603 (25.7) | 1 (1-2) | 4502 (2304—6161) |
|  | 10000-99999 | 27 (14.6) | 55 | 847148 (52.7) | 1 (1-3) | 25160 (13950—34505) |
|  | ≥ 100000 | 2 (1.1) | 41 | 327539 (20.4) | 20 | 163769 |
| **Ownership** | <1000 | 2 (12.5) | 2 | 10 (<0.1) | 2 | 5 |
|  | 1000-9999 | 2 (12.5) | 4 | 6085 (0.1) | 2 | 3043 |
|  | 10000-99999 | 10 (62.5) | 10 | 397904 (11.9) | 1 | 30653 (25000—50000) |
|  | ≥ 100000 | 2 (12.5) | 3 | 2937663 (87.9) | 1.5 | 1468831.50 |

**Supplemental Table 2: General Payments: Top 10 Aggregated payments per physician by Drug or Device manufacturers**

| **Name of the manufacturer** | **Compensation for service** | **Consulting fees** | **Education** | **Food** | **Travel** | **Others^§^** |
| --- | --- | --- | --- | --- | --- | --- |
| Abbvie | 14801 | 6282 | 26 | 209 | 2265 | 1569 (Grant) |
| Gilead sciences | 18521 | 5714 | 49 | 150 | 2674 | 114081 (Grant) |
| Allergan | 9852 | 691 | 21 | 138 | 1873 | 20 (Gift) |
| Valeant pharmaceuticals | 2509 | 10392 | 20 | 134 | 2119 | 12829 (Compensation for serving)  96 (Gift)  113392 (Royalty) |
| Takeda* | 14266 | 20110 | 301 | 78 | 2803 |  |
| Merck Sharp & Dohme Corporation | 14753 | 6529 | 351 | 67 | 2861 |  |
| Cook* | 7426 | 25555 | 21200 | 118 | 774 | 964 (Compensation for serving)  87034 (Grant)  1784517 (Royalty) |
| Johnson & Johnson* | 19963 | 4398 | 79 | 97 | 3376 |  |
| Braintree Laboratories | 45001 | 8507 |  | 29 | 2562 | 967 (Grant)  1818511 (Royalty) |
| Boston Scientific Corporation | 8854 | 4632 | 409 | 114 | 874 | 97138 (Current or prospective)  1070 (Grant) |
